# Atrial and placental melanoma metastasis: a case report and literature review

**DOI:** 10.1186/1752-1947-1-21

**Published:** 2007-05-14

**Authors:** Pradeep Lakshminarayana, Sarah Danson, Kim Suvarna, Barry Hancock

**Affiliations:** 1 Department of Clinical Oncology, Weston Park Hospital, Whitham Road, Sheffield, S10 2SJ, UK; 2 Department of Histopathology, Northern General Hospital, Herries Road, Sheffield, S5 7AU, UK

## Abstract

Malignant melanoma can metastasize to virtually any organ of the body. The aggressiveness is determined by the primary site, depth of dermal invasion, presence or absence of ulceration, lymphovascular infiltration and regional lymph node involvement. We report a case of a pregnant woman with a previous history of stage 3 melanoma who presented with cardiac metastasis and placental melanoma infiltration. A review of literature on cardiac and placental involvement of melanoma is also provided.

## Background

Malignant melanoma is considered to be a highly aggressive cancer. Post mortem studies have shown that melanoma can metastasize to any organ of the body, with 50% of the patients found to have cardiac involvement [1]. However only 2% of patients with metastatic melanoma to the heart are diagnosed antemortem [2], as most patients do not have any cardiac symptoms. Melanoma is also unique in that it is the most common cancer to metastasize to the placenta [3].

We report a pregnant woman with invasive melanoma who presented with metastasis in the right atrium and placental melanoma infiltration. The balance of surgery, chemotherapy and obstetric management in this case is challenging.

## Case report

A 32 year old woman, initially presented at the age of 27 with stage 3 malignant melanoma. The patient underwent wide local excision of a 1.76 mm Breslow thickness melanoma from the right thigh and shortly afterwards was found to have a mass in the right groin. She underwent right inguinal block dissection, with 2 of 6 lymph nodes positive for melanoma. CT scan showed no evidence of other disease sites. The patient had adjuvant high dose interferon (20 MU/m^2^/d IV 5 days/wk × 4 weeks followed by 10 MU/m^2 ^SC thrice weekly × 48 weeks) which was discontinued after nearly 7 months due to persistent neutropenia.

When aged 31 and 19 weeks pregnant, the patient noticed a mass beneath her jaw consistent with right submandibular lymph gland enlargement and had exertional dypsnoea. A CT scan revealed a pericardial effusion of 1 cm depth. A transthoracic echocardiogram showed a right atrial mass prolapsing through the tricuspid valve with good preservation of ventricular function.

**Figure 1:**
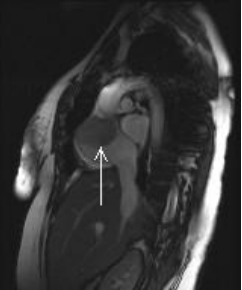
MRI of the heart showing low signal density in the right atrial (arrowed) representing metastasis.

**Figure 2:**
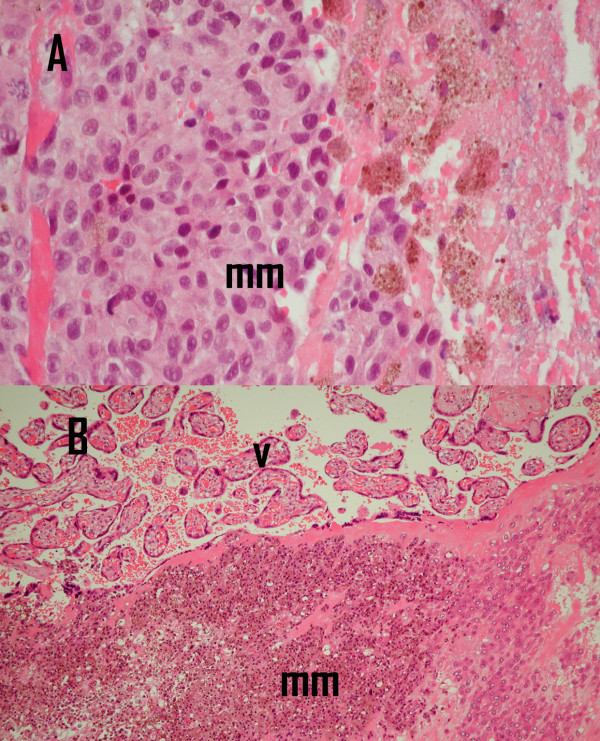
**Histopathology: A. **Endomyocardial biopsy showed pleomorphic malignant melanoma(mm) with associated necrosis (original magnification × 200). **B. **Placental tissue shows villous tissue(v) with foci of malignant melanoma(mm) (original magnification × 200).

An MRI scan of the heart confirmed the mass to be more extensive measuring 8 cm in size and extending into the superior vena cava (fig 1). An echocardiogram guided biopsy of the mass (fig 2A) revealed necrotic tumour with associated thrombus and adjacent normal myocardium. The viable tumour was composed of moderately pleomorphic epitheliod melanocyes (S100/HMB45 positive) with enlarged pleomorphic nuclei and prominent nucleoli.

The patient was keen to continue with pregnancy and after careful consideration was commenced on palliative chemotherapy with dacarbazine (800 mg/m^2 ^IV every 3 weeks). The decision was to deliver the baby early by elective Caesarean section at 34 weeks of gestation considering the risk of sudden cardiac death of the mother, which would likely be associated with fetal demise. After 2 cycles of dacarbazine, an echocardiogram showed that the atrial mass was smaller.

However an emergency Caesarean section was performed at 31 weeks gestation because of ante-partum haemorrhage. The baby delivered weighed 1.5 kg with no significant problems in the neonatal period. Histological examination of the placental tissue showed unremarkable cord and membranes. The placental bed showed an early third trimester architecture with good villous development. Focally there were groups of viable epitheliod melanocytes, similar to the cardiac tissues with associated thrombus (fig 2B). Immunohistochemistry revealed S100, HMB45 and melan A positivity, consistent with melanoma. As there had been a response on the echocardiogram, we decided to proceed to 8 cycles of dacarbazine chemotherapy. Following this, an MRI scan of the heart showed less tumour bulk with a maximum dimension of less than 2 × 3 cm. Clinically the patient had no dyspnoea on exertion. The right mandibular lymph gland enlargement was persistent and a selective neck dissection was done. This showed central degeneration in one lymph node with a peripheral rim of fibroconnective tissue showing melanin deposition, representing a response to chemotherapy.

Despite the metastatic nature of her disease, this patient remains well and is currently awaiting opinion for cardiac surgery. Her baby is now 10 months old and is free of disease.

## Discussion

Metastatic melanoma of the heart is generally a part of widespread tumour dissemination. The surgical management of these patients varies depending on presence or absence of extracardiac involvement and also the extent of the cardiac tumour. If anatomically feasible, complete resection with reconstruction should be attempted [4]. Indications for complete surgical resection are patients with a preoperative Karnofsky performance status of more than 80%, minimal extracardiac disease, or a deteriorating clinical picture due to cardiac symptoms [4]. In extensive disease, surgical debulking may be considered as a palliative therapeutic option.

Chemotherapy was considered to be the best available option of treatment in this case, considering the risk of a major surgical procedure on the viability of the fetus. There are some previously published reports of the use of chemotherapy for metastatic melanoma in pregnancy. Harkin et al. reported the use of dacarbazine in the 3^rd ^trimester which induced dramatic remission [5]. Another report suggested that combination chemotherapy which included dacarbazine when used in the 2^nd ^trimester did not interfere with the maturation and delivery of the infant [6]. No congenital anomalies in infants born to women treated with dacarbazine have been reported after treatment during the 2^nd ^or 3^rd ^trimester.

Sorafenib an oral targeted agent and a multi-kinase inhibitor, is well tolerated but has little or no anti tumour activity when used alone in advanced melanoma [7]. Ongoing trials in advanced melanoma are evaluating sorafenib in combination therapies. However this agent has not been studied in pregnant women and is not recommended for use in pregnancy.

Placental and fetal metastasis related to cancers have been documented before, the most common being melanoma [8]. The risk of development of melanoma in the fetus with placental involvement is approximately 22%. Babies born with placental metastasis should therefore be considered high risk and monitored closely. Infants developing clinical evidence of maternally derived metastasis have poor prognosis with mortality reaching 100% and death typically occurring within 3 months of diagnosis [8].

Some difficult ethical issues arise from this case. Firstly, because of poor median survival of 6 months in pregnant women with metastatic melanoma which may make them seriously ill before term, putting the life of the fetus at risk [9]. However, recent reports suggest that there is no significant difference in outcome and survival rate between pregnant and non-pregnant women with melanoma [10]. Secondly, there is an increased risk of melanoma in the infant and when found the prognosis is invariably poor. Maternal factors associated with an unfavourable infant prognosis include maternal age less than 30 years, primiparity, disease onset more than 3 years before current pregnancy, nodal metastasis before pregnancy, more than three sites of metastatic foci during the third trimester, primary site of the leg, and maternal death within 1 month of birth [3]. Counselling of pregnant women who develop metastatic melanoma is vital. The patient and carers need support in their decisions about whether to continue with pregnancy and which treatment options to pursue.

## Conclusion

Metastatic melanoma can sometimes be associated with a good outcome as illustrated in this case. Chemotherapy is a reasonable alternative to surgery when used for disease control in an obstetric setting. A multidisciplinary team approach is vital in management of these complex cases.

## Competing interests

The author(s) declare that they have no competing interests.

## Authors' contributions

PL is primarily responsible for drafting, literature search, submission and revision of the manuscript. SD and BH are responsible for manuscript editing and advice on literature review. KS is responsible for writing up pathology, providing the slides and manuscript editing. All authors have read and approved the final manuscript.
